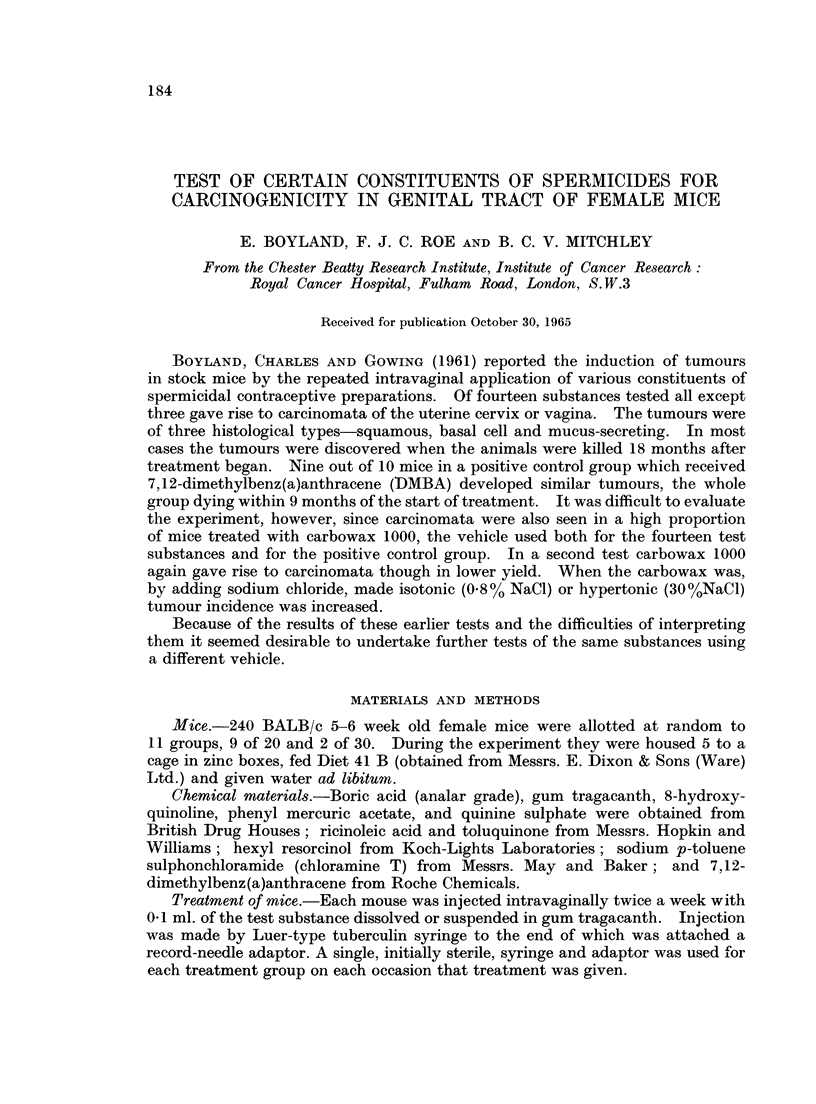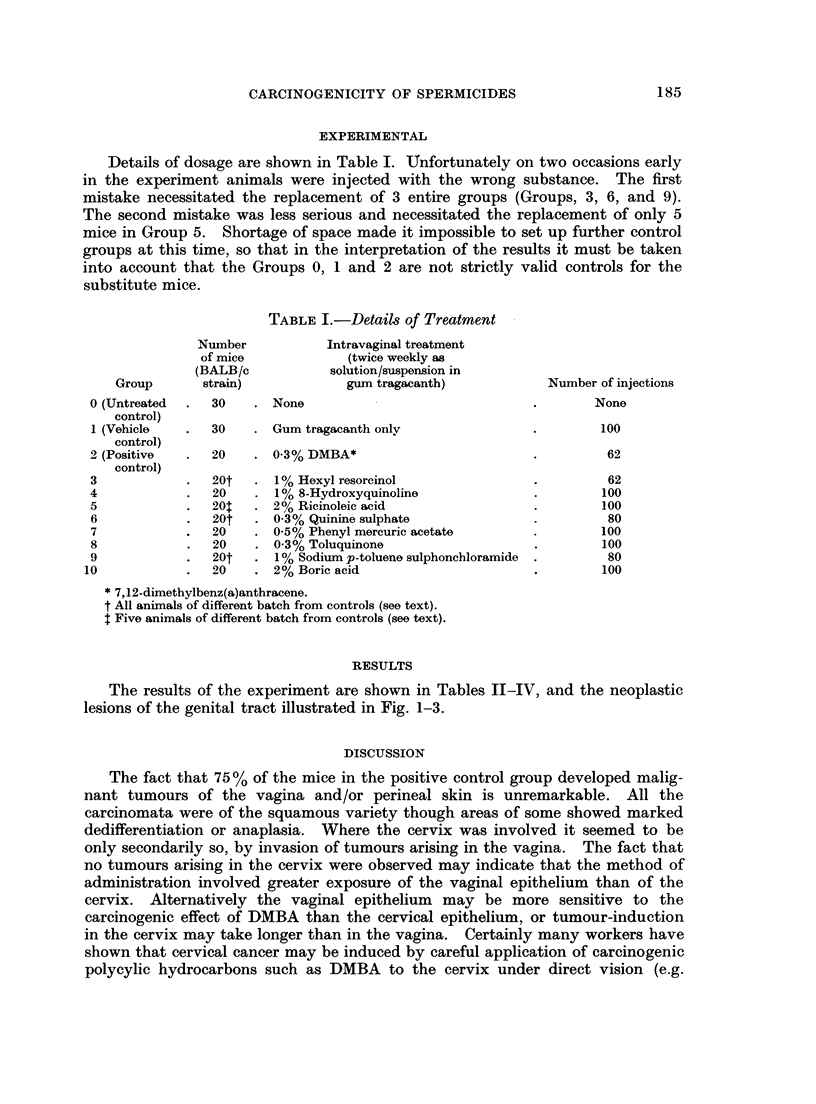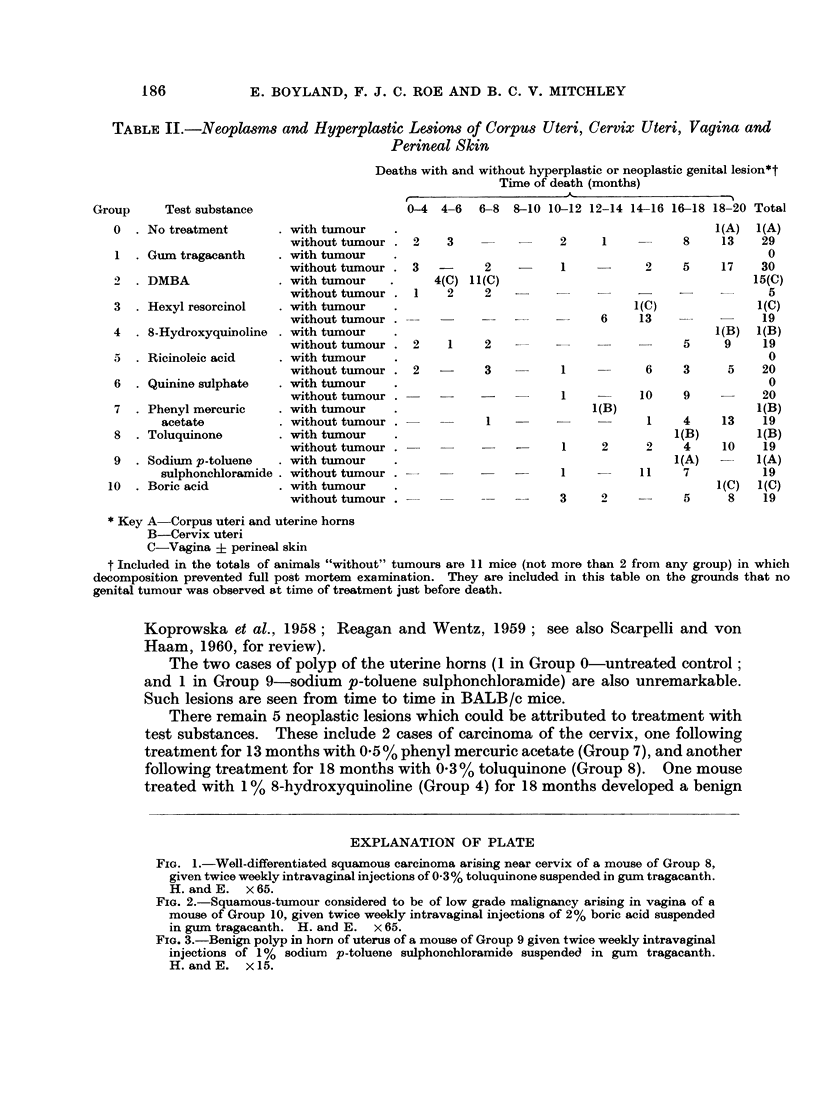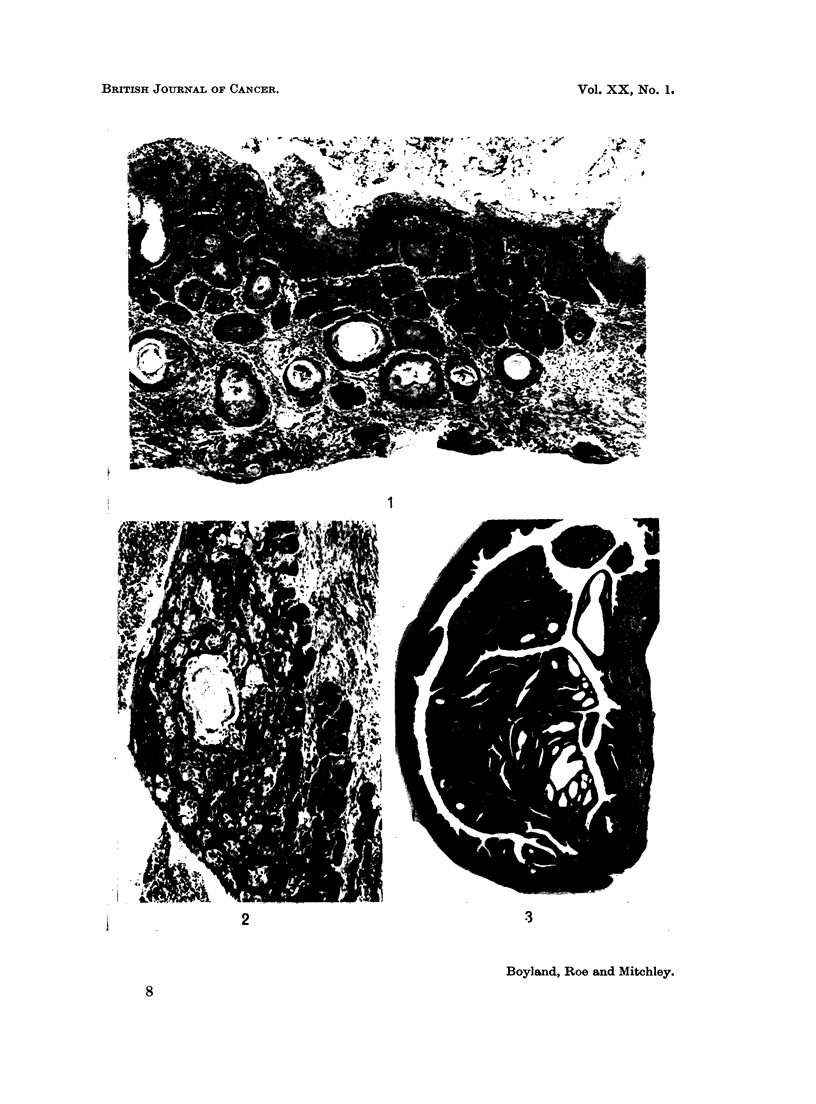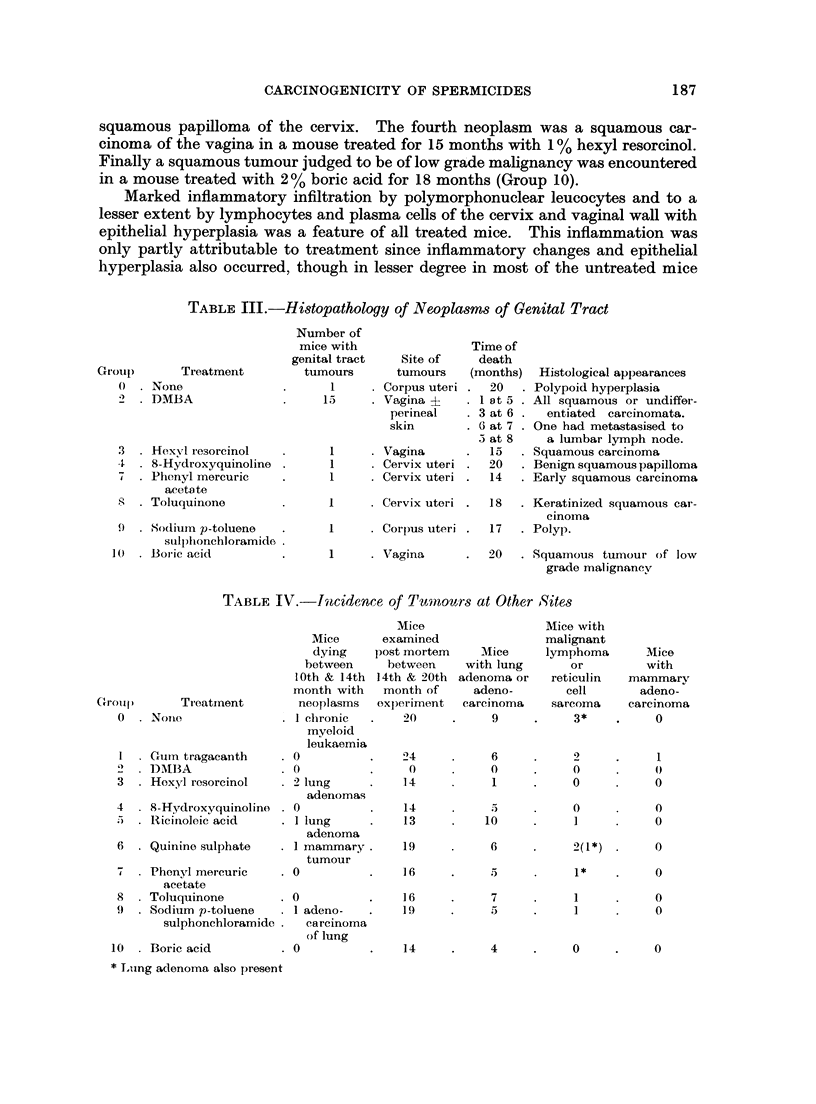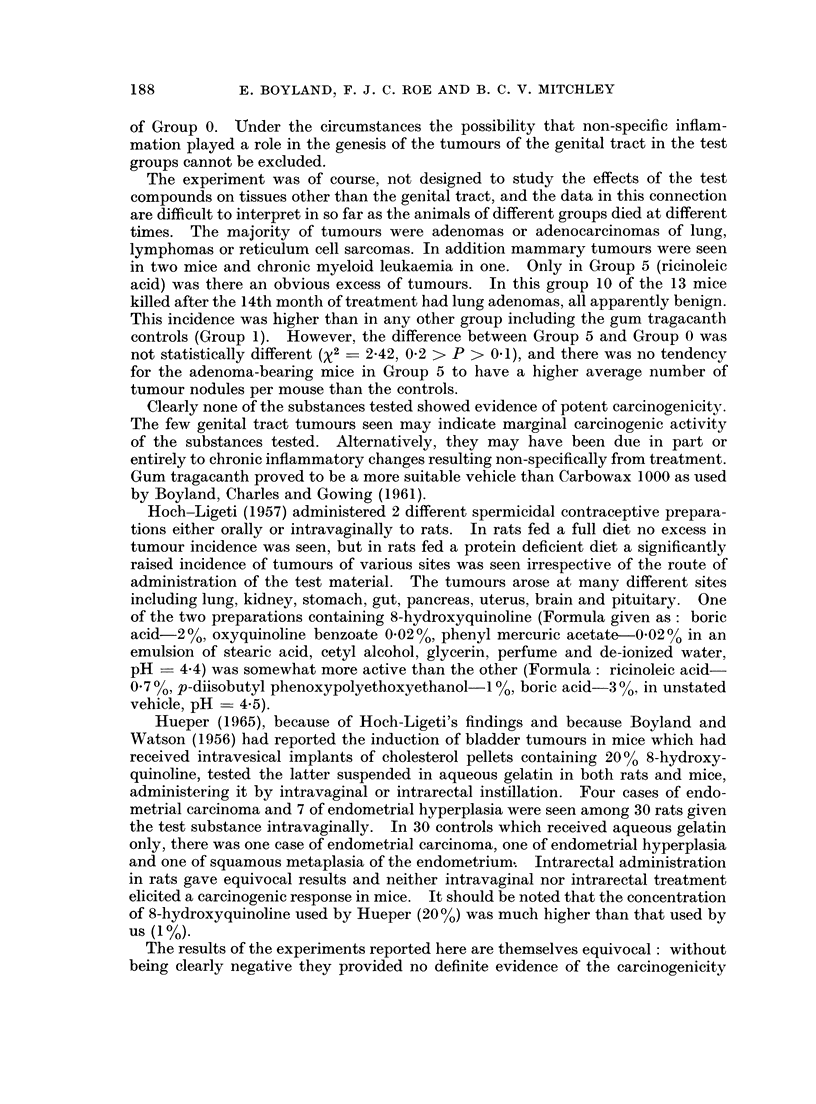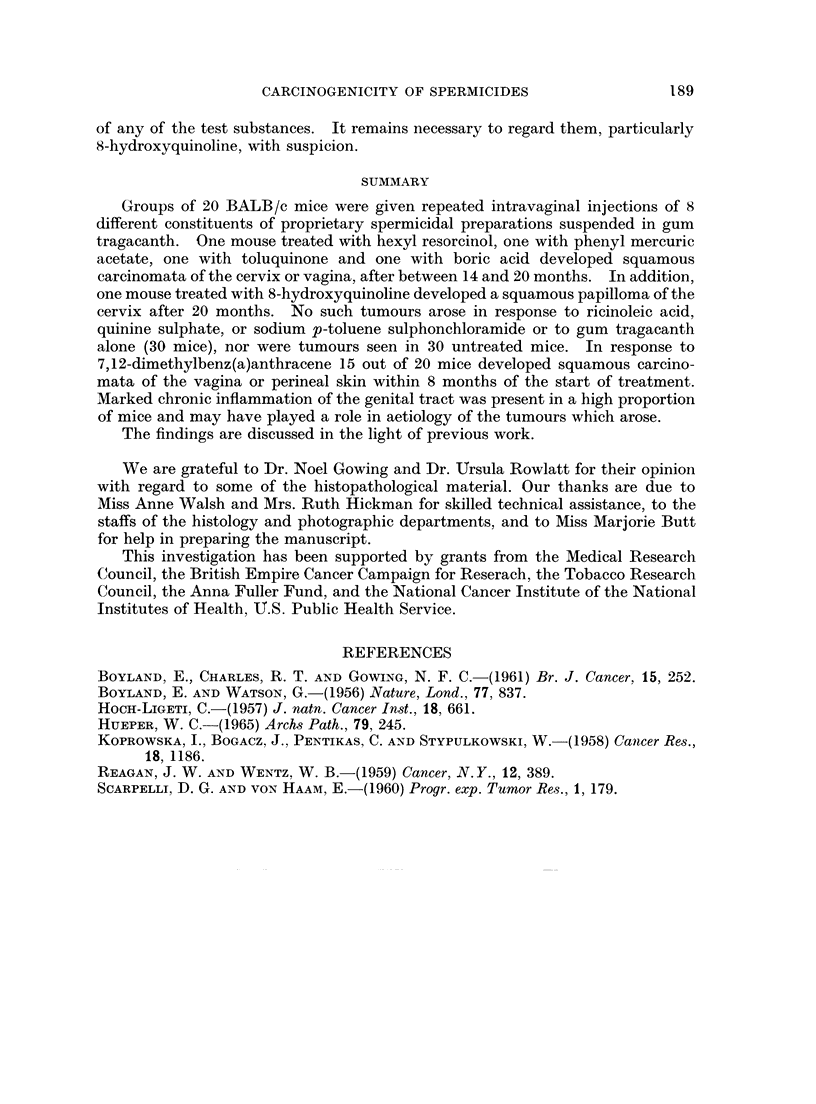# Test of certain constituents of spermicides for carcinogenicity in genital tract of female mice.

**DOI:** 10.1038/bjc.1966.21

**Published:** 1966-03

**Authors:** E. Boyland, F. J. Roe, B. C. Mitchley

## Abstract

**Images:**


### TEST OF CERTAIN CONSTITUENTS OF SPERMICIDES FOR CARCINOGENICITY IN GENITAL TRACT OF FEMALE MICE

E. BOYLAND, F. J. C. ROE AND B. C. V. MITCHLEY

*From the Chester Beatty Research Institute, Institute of Cancer Research : Royal Cancer Hospital, Fulham Road, London, S.W.3*

Received for publication October 30, 1965

BOYLAND, CHARLES AND GOWING (1961) reported the induction of tumours in stock mice by the repeated intravaginal application of various constituents of spermicidal contraceptive preparations. Of fourteen substances tested all except three gave rise to carcinomata of the uterine cervix or vagina. The tumours were of three histological types—squamous, basal cell and mucus-secreting. In most cases the tumours were discovered when the animals were killed 18 months after treatment began. Nine out of 10 mice in a positive control group which received 7,12-dimethylbenz(a)anthracene (DMBA) developed similar tumours, the whole group dying within 9 months of the start of treatment. It was difficult to evaluate the experiment, however, since carcinomata were also seen in a high proportion of mice treated with carbowax 1000, the vehicle used both for the fourteen test substances and for the positive control group. In a second test carbowax 1000 again gave rise to carcinomata though in lower yield. When the carbowax was, by adding sodium chloride, made isotonic (0·8% NaCl) or hypertonic (30%NaCl) tumour incidence was increased.

Because of the results of these earlier tests and the difficulties of interpreting them it seemed desirable to undertake further tests of the same substances using a different vehicle.

### MATERIALS AND METHODS

*Mice.*—240 BALB/c 5–6 week old female mice were allotted at random to 11 groups, 9 of 20 and 2 of 30. During the experiment they were housed 5 to a cage in zinc boxes, fed Diet 41 B (obtained from Messrs. E. Dixon & Sons (Ware) Ltd.) and given water *ad libitum.*

*Chemical materials.*—Boric acid (analar grade), gum tragacanth, 8-hydroxyquinoline, phenyl mercuric acetate, and quinine sulphate were obtained from British Drug Houses ; ricinoleic acid and toluquinone from Messrs. Hopkin and Williams ; hexyl resorcinol from Koch-Lights Laboratories ; sodium *p*-toluene sulphonchloramide (chloramine T) from Messrs. May and Baker ; and 7,12-dimethylbenz(a)anthracene from Roche Chemicals.

*Treatment of mice.*—Each mouse was injected intravaginally twice a week with 0·1 ml. of the test substance dissolved or suspended in gum tragacanth. Injection was made by Luer-type tuberculin syringe to the end of which was attached a record-needle adaptor. A single, initially sterile, syringe and adaptor was used for each treatment group on each occasion that treatment was given.

### EXPERIMENTAL

Details of dosage are shown in Table I. Unfortunately on two occasions early in the experiment animals were injected with the wrong substance. The first mistake necessitated the replacement of 3 entire groups (Groups, 3, 6, and 9). The second mistake was less serious and necessitated the replacement of only 5 mice in Group 5. Shortage of space made it impossible to set up further control groups at this time, so that in the interpretation of the results it must be taken into account that the Groups 0, 1 and 2 are not strictly valid controls for the substitute mice.

TABLE I.—*Details of Treatment*

| Group | Number of mice (BALB/c strain) | Intravaginal treatment (twice weekly as solution/suspension in gum tragacanth) | Number of injections |
|---|---|---|---|
| 0 (Untreated control) | 30 | None | None |
| 1 (Vehicle control) | 30 | Gum tragacanth only | 100 |
| 2 (Positive control) | 20 | 0·3% DMBA* | 62 |
| 3 | 20† | 1% Hexyl resorcinol | 62 |
| 4 | 20 | 1% 8-Hydroxyquinoline | 100 |
| 5 | 20‡ | 2% Ricinoleic acid | 100 |
| 6 | 20† | 0·3% Quinine sulphate | 80 |
| 7 | 20 | 0·5% Phenyl mercuric acetate | 100 |
| 8 | 20 | 0·3% Toluquinone | 100 |
| 9 | 20† | 1% Sodium *p*-toluene sulphonchloramide | 80 |
| 10 | 20 | 2% Boric acid | 100 |

* 7,12-dimethylbenz(a)anthracene.
† All animals of different batch from controls (see text).
‡ Five animals of different batch from controls (see text).

### RESULTS

The results of the experiment are shown in Tables II–IV, and the neoplastic lesions of the genital tract illustrated in Fig. 1–3.

### DISCUSSION

The fact that 75% of the mice in the positive control group developed malignant tumours of the vagina and/or perineal skin is unremarkable. All the carcinomata were of the squamous variety though areas of some showed marked dedifferentiation or anaplasia. Where the cervix was involved it seemed to be only secondarily so, by invasion of tumours arising in the vagina. The fact that no tumours arising in the cervix were observed may indicate that the method of administration involved greater exposure of the vaginal epithelium than of the cervix. Alternatively the vaginal epithelium may be more sensitive to the carcinogenic effect of DMBA than the cervical epithelium, or tumour-induction in the cervix may take longer than in the vagina. Certainly many workers have shown that cervical cancer may be induced by careful application of carcinogenic polycylic hydrocarbons such as DMBA to the cervix under direct vision (e.g.

TABLE II.—*Neoplasms and Hyperplastic Lesions of Corpus Uteri, Cervix Uteri, Vagina and Perineal Skin*

| Group | Test substance | | Deaths with and without hyperplastic or neoplastic genital lesion*† Time of death (months) 0–4 | 4–6 | 6–8 | 8–10 | 10–12 | 12–14 | 14–16 | 16–18 | 18–20 | Total |
|---|---|---|---|---|---|---|---|---|---|---|---|---|
| 0 | No treatment | with tumour | | | | | | | | | 1(A) | 1(A) |
| | | without tumour | 2 | 3 | — | — | 2 | 1 | — | 8 | 13 | 29 |
| 1 | Gum tragacanth | with tumour | | | | | | | | | | 0 |
| | | without tumour | 3 | — | 2 | — | 1 | — | 2 | 5 | 17 | 30 |
| 2 | DMBA | with tumour | | 4(C) | 11(C) | | | | | | | 15(C) |
| | | without tumour | 1 | 2 | 2 | — | — | — | — | — | — | 5 |
| 3 | Hexyl resorcinol | with tumour | | | | | | | 1(C) | | | 1(C) |
| | | without tumour | — | — | — | — | — | 6 | 13 | — | — | 19 |
| 4 | 8-Hydroxyquinoline | with tumour | | | | | | | | | 1(B) | 1(B) |
| | | without tumour | 2 | 1 | 2 | — | — | — | — | 5 | 9 | 19 |
| 5 | Ricinoleic acid | with tumour | | | | | | | | | | 0 |
| | | without tumour | 2 | — | 3 | — | 1 | — | 6 | 3 | 5 | 20 |
| 6 | Quinine sulphate | with tumour | | | | | | | | | | 0 |
| | | without tumour | — | — | — | — | 1 | — | 10 | 9 | — | 20 |
| 7 | Phenyl mercuric | with tumour | | | | | | 1(B) | | | | 1(B) |
| | acetate | without tumour | — | — | 1 | — | — | — | 1 | 4 | 13 | 19 |
| 8 | Toluquinone | with tumour | | | | | | | | 1(B) | | 1(B) |
| | | without tumour | — | — | — | — | 1 | 2 | 2 | 4 | 10 | 19 |
| 9 | Sodium *p*-toluene | with tumour | | | | | | | | 1(A) | — | 1(A) |
| | sulphonchloramide | without tumour | — | — | — | — | 1 | — | 11 | 7 | | 19 |
| 10 | Boric acid | with tumour | | | | | | | | | 1(C) | 1(C) |
| | | without tumour | — | — | — | — | 3 | 2 | — | 5 | 8 | 19 |

* Key A—Corpus uteri and uterine horns
B—Cervix uteri
C—Vagina ± perineal skin

† Included in the totals of animals "without" tumours are 11 mice (not more than 2 from any group) in which decomposition prevented full post mortem examination. They are included in this table on the grounds that no genital tumour was observed at time of treatment just before death.

Koprowska *et al.*, 1958 ; Reagan and Wentz, 1959 ; see also Scarpelli and von Haam, 1960, for review).

The two cases of polyp of the uterine horns (1 in Group 0—untreated control ; and 1 in Group 9—sodium *p*-toluene sulphonchloramide) are also unremarkable. Such lesions are seen from time to time in BALB/c mice.

There remain 5 neoplastic lesions which could be attributed to treatment with test substances. These include 2 cases of carcinoma of the cervix, one following treatment for 13 months with 0·5% phenyl mercuric acetate (Group 7), and another following treatment for 18 months with 0·3% toluquinone (Group 8). One mouse treated with 1% 8-hydroxyquinoline (Group 4) for 18 months developed a benign

---

EXPLANATION OF PLATE

FIG. 1.—Well-differentiated squamous carcinoma arising near cervix of a mouse of Group 8, given twice weekly intravaginal injections of 0·3% toluquinone suspended in gum tragacanth. H. and E. ×65.

FIG. 2.—Squamous-tumour considered to be of low grade malignancy arising in vagina of a mouse of Group 10, given twice weekly intravaginal injections of 2% boric acid suspended in gum tragacanth. H. and E. ×65.

FIG. 3.—Benign polyp in horn of uterus of a mouse of Group 9 given twice weekly intravaginal injections of 1% sodium *p*-toluene sulphonchloramide suspended in gum tragacanth. H. and E. ×15.

1

2

3

squamous papilloma of the cervix. The fourth neoplasm was a squamous carcinoma of the vagina in a mouse treated for 15 months with 1% hexyl resorcinol. Finally a squamous tumour judged to be of low grade malignancy was encountered in a mouse treated with 2% boric acid for 18 months (Group 10).

Marked inflammatory infiltration by polymorphonuclear leucocytes and to a lesser extent by lymphocytes and plasma cells of the cervix and vaginal wall with epithelial hyperplasia was a feature of all treated mice. This inflammation was only partly attributable to treatment since inflammatory changes and epithelial hyperplasia also occurred, though in lesser degree in most of the untreated mice

TABLE III.—*Histopathology of Neoplasms of Genital Tract*

| Group | Treatment | Number of mice with genital tract tumours | Site of tumours | Time of death (months) | Histological appearances |
|---|---|---|---|---|---|
| 0 | None | 1 | Corpus uteri | 20 | Polypoid hyperplasia |
| 2 | DMBA | 15 | Vagina ± perineal skin | 1 at 5<br>3 at 6<br>6 at 7<br>5 at 8 | All squamous or undifferentiated carcinomata. One had metastasised to a lumbar lymph node. |
| 3 | Hexyl resorcinol | 1 | Vagina | 15 | Squamous carcinoma |
| 4 | 8-Hydroxyquinoline | 1 | Cervix uteri | 20 | Benign squamous papilloma |
| 7 | Phenyl mercuric acetate | 1 | Cervix uteri | 14 | Early squamous carcinoma |
| 8 | Toluquinone | 1 | Cervix uteri | 18 | Keratinized squamous carcinoma |
| 9 | Sodium *p*-toluene sulphonchloramide | 1 | Corpus uteri | 17 | Polyp. |
| 10 | Boric acid | 1 | Vagina | 20 | Squamous tumour of low grade malignancy |

TABLE IV.—*Incidence of Tumours at Other Sites*

| Group | Treatment | Mice dying between 10th & 14th month with neoplasms | Mice examined post mortem between 14th & 20th month of experiment | Mice with lung adenoma or adeno-carcinoma | Mice with malignant lymphoma or reticulin cell sarcoma | Mice with mammary adeno-carcinoma |
|---|---|---|---|---|---|---|
| 0 | None | 1 chronic myeloid leukaemia | 20 | 9 | 3* | 0 |
| 1 | Gum tragacanth | 0 | 24 | 6 | 2 | 1 |
| 2 | DMBA | 0 | 0 | 0 | 0 | 0 |
| 3 | Hexyl resorcinol | 2 lung adenomas | 14 | 1 | 0 | 0 |
| 4 | 8-Hydroxyquinoline | 0 | 14 | 5 | 0 | 0 |
| 5 | Ricinoleic acid | 1 lung adenoma | 13 | 10 | 1 | 0 |
| 6 | Quinine sulphate | 1 mammary tumour | 19 | 6 | 2(1*) | 0 |
| 7 | Phenyl mercuric acetate | 0 | 16 | 5 | 1* | 0 |
| 8 | Toluquinone | 0 | 16 | 7 | 1 | 0 |
| 9 | Sodium *p*-toluene sulphonchloramide | 1 adeno-carcinoma of lung | 19 | 5 | 1 | 0 |
| 10 | Boric acid | 0 | 14 | 4 | 0 | 0 |

* Lung adenoma also present of Group 0. Under the circumstances the possibility that non-specific inflammation played a role in the genesis of the tumours of the genital tract in the test groups cannot be excluded.

The experiment was of course, not designed to study the effects of the test compounds on tissues other than the genital tract, and the data in this connection are difficult to interpret in so far as the animals of different groups died at different times. The majority of tumours were adenomas or adenocarcinomas of lung, lymphomas or reticulum cell sarcomas. In addition mammary tumours were seen in two mice and chronic myeloid leukaemia in one. Only in Group 5 (ricinoleic acid) was there an obvious excess of tumours. In this group 10 of the 13 mice killed after the 14th month of treatment had lung adenomas, all apparently benign. This incidence was higher than in any other group including the gum tragacanth controls (Group 1). However, the difference between Group 5 and Group 0 was not statistically different ($\chi^2 = 2{\cdot}42$, $0{\cdot}2 > P > 0{\cdot}1$), and there was no tendency for the adenoma-bearing mice in Group 5 to have a higher average number of tumour nodules per mouse than the controls.

Clearly none of the substances tested showed evidence of potent carcinogenicity. The few genital tract tumours seen may indicate marginal carcinogenic activity of the substances tested. Alternatively, they may have been due in part or entirely to chronic inflammatory changes resulting non-specifically from treatment. Gum tragacanth proved to be a more suitable vehicle than Carbowax 1000 as used by Boyland, Charles and Gowing (1961).

Hoch–Ligeti (1957) administered 2 different spermicidal contraceptive preparations either orally or intravaginally to rats. In rats fed a full diet no excess in tumour incidence was seen, but in rats fed a protein deficient diet a significantly raised incidence of tumours of various sites was seen irrespective of the route of administration of the test material. The tumours arose at many different sites including lung, kidney, stomach, gut, pancreas, uterus, brain and pituitary. One of the two preparations containing 8-hydroxyquinoline (Formula given as : boric acid—2%, oxyquinoline benzoate 0·02%, phenyl mercuric acetate—0·02% in an emulsion of stearic acid, cetyl alcohol, glycerin, perfume and de-ionized water, pH = 4·4) was somewhat more active than the other (Formula : ricinoleic acid—0·7%, *p*-diisobutyl phenoxypolyethoxyethanol—1%, boric acid—3%, in unstated vehicle, pH = 4·5).

Hueper (1965), because of Hoch-Ligeti's findings and because Boyland and Watson (1956) had reported the induction of bladder tumours in mice which had received intravesical implants of cholesterol pellets containing 20% 8-hydroxyquinoline, tested the latter suspended in aqueous gelatin in both rats and mice, administering it by intravaginal or intrarectal instillation. Four cases of endometrial carcinoma and 7 of endometrial hyperplasia were seen among 30 rats given the test substance intravaginally. In 30 controls which received aqueous gelatin only, there was one case of endometrial carcinoma, one of endometrial hyperplasia and one of squamous metaplasia of the endometrium. Intrarectal administration in rats gave equivocal results and neither intravaginal nor intrarectal treatment elicited a carcinogenic response in mice. It should be noted that the concentration of 8-hydroxyquinoline used by Hueper (20%) was much higher than that used by us (1%).

The results of the experiments reported here are themselves equivocal : without being clearly negative they provided no definite evidence of the carcinogenicity of any of the test substances. It remains necessary to regard them, particularly 8-hydroxyquinoline, with suspicion.

SUMMARY

Groups of 20 BALB/c mice were given repeated intravaginal injections of 8 different constituents of proprietary spermicidal preparations suspended in gum tragacanth. One mouse treated with hexyl resorcinol, one with phenyl mercuric acetate, one with toluquinone and one with boric acid developed squamous carcinomata of the cervix or vagina, after between 14 and 20 months. In addition, one mouse treated with 8-hydroxyquinoline developed a squamous papilloma of the cervix after 20 months. No such tumours arose in response to ricinoleic acid, quinine sulphate, or sodium *p*-toluene sulphonchloramide or to gum tragacanth alone (30 mice), nor were tumours seen in 30 untreated mice. In response to 7,12-dimethylbenz(a)anthracene 15 out of 20 mice developed squamous carcinomata of the vagina or perineal skin within 8 months of the start of treatment. Marked chronic inflammation of the genital tract was present in a high proportion of mice and may have played a role in aetiology of the tumours which arose.

The findings are discussed in the light of previous work.

We are grateful to Dr. Noel Gowing and Dr. Ursula Rowlatt for their opinion with regard to some of the histopathological material. Our thanks are due to Miss Anne Walsh and Mrs. Ruth Hickman for skilled technical assistance, to the staffs of the histology and photographic departments, and to Miss Marjorie Butt for help in preparing the manuscript.

This investigation has been supported by grants from the Medical Research Council, the British Empire Cancer Campaign for Reserach, the Tobacco Research Council, the Anna Fuller Fund, and the National Cancer Institute of the National Institutes of Health, U.S. Public Health Service.

### REFERENCES

BOYLAND, E., CHARLES, R. T. AND GOWING, N. F. C.—(1961) *Br. J. Cancer*, **15**, 252.
BOYLAND, E. AND WATSON, G.—(1956) *Nature, Lond.*, **77**, 837.
HOCH-LIGETI, C.—(1957) *J. natn. Cancer Inst.*, **18**, 661.
HUEPER, W. C.—(1965) *Archs Path.*, **79**, 245.
KOPROWSKA, I., BOGACZ, J., PENTIKAS, C. AND STYPULKOWSKI, W.—(1958) *Cancer Res.*, **18**, 1186.
REAGAN, J. W. AND WENTZ, W. B.—(1959) *Cancer, N.Y.*, **12**, 389.
SCARPELLI, D. G. AND VON HAAM, E.—(1960) *Progr. exp. Tumor Res.*, **1**, 179.